# The Mystery of an Eviscerated Bowel With a Yellow Tube: A Rare Presentation of Feeding Jejunostomy Intussusception

**DOI:** 10.7759/cureus.105798

**Published:** 2026-03-24

**Authors:** Neel Swaminarayan

**Affiliations:** 1 Department of General Surgery, Government Medical College and New Civil Hospital, Surat, IND; 2 Department of Urology, Dr. Baba Saheb Ambedkar Hospital, New Delhi, IND

**Keywords:** abdominal surgery complications, distal enteral feeding, end-to-end anastomosis, feeding jejunostomy, general emergency surgery, jejunojejunal intussusception, surgical gastroenterology

## Abstract

Feeding jejunostomy (FJ) is a standard surgical procedure for establishing long-term enteral access. While minor site complications are common, severe mechanical disruptions are exceedingly rare. We report a highly unusual and dramatic presentation of an FJ-induced complication. A 28-year-old male patient presented to the emergency department with a striking clinical picture: an eviscerated, congested rosette of bowel with a "yellow tube" protruding directly from the prolapsed bowel segment. Emergency exploratory laparotomy revealed a complex jejuno-jejunal intussusception acting as a lead point at the previous FJ site, further complicated by a localized intestinal perforation. The patient underwent a limited resection of the compromised jejunal segment followed by a primary end-to-end anastomosis. To maintain nutritional access, a new FJ was established distally in the healthy bowel. The postoperative course was completely uneventful, and the patient was discharged on postoperative day 5. This case highlights an exceptionally rare, life-threatening, and preventable mechanical complication of FJs. An eviscerated, intussuscepted bowel with concurrent perforation demands immediate surgical exploration, resection of the ischemic segment, and re-establishment of distal enteral feeding to ensure a successful recovery.

## Introduction

Feeding jejunostomy (FJ) is a well-established and life-saving surgical intervention for providing long-term enteral nutrition in patients who cannot tolerate oral or gastric feeding. While generally considered a safe procedure, it carries a reported overall complication rate ranging from 2% to 12% [1]. The majority of these complications are minor and easily manageable, including tube blockade, accidental dislodgement, peri-tubal leakage, and localized skin infections.

However, major mechanical complications, such as small bowel obstruction, volvulus, ischemia, and intussusception, are exceedingly rare and potentially fatal if not recognized promptly [2]. Adult intussusception itself is an uncommon clinical entity, accounting for only 1-5% of all cases of mechanical bowel obstruction [3]. Unlike pediatric populations, where the cause is often idiopathic, adult intussusception typically has a definable structural lesion acting as a lead point in 70-90% of cases [4]. In the context of an FJ, the tube itself can inadvertently act as this mechanical lead point, altering normal antegrade peristalsis and causing the jejunal wall to telescope distally.

We report the case of a 28-year-old man presenting with an "eviscerated bowel with a yellow tube," highlighting the underlying pathophysiology, the necessity of urgent surgical exploration, and the principles of managing this severe mechanical complication.

## Case presentation

A 28-year-old male patient presented to the surgical emergency department with a sudden and dramatic protrusion of bowel from his abdomen. His surgical history was significant for an FJ created previously for enteral nutrition, for an esophageal stricture due to corrosive ingestion.

**Figure 1:**
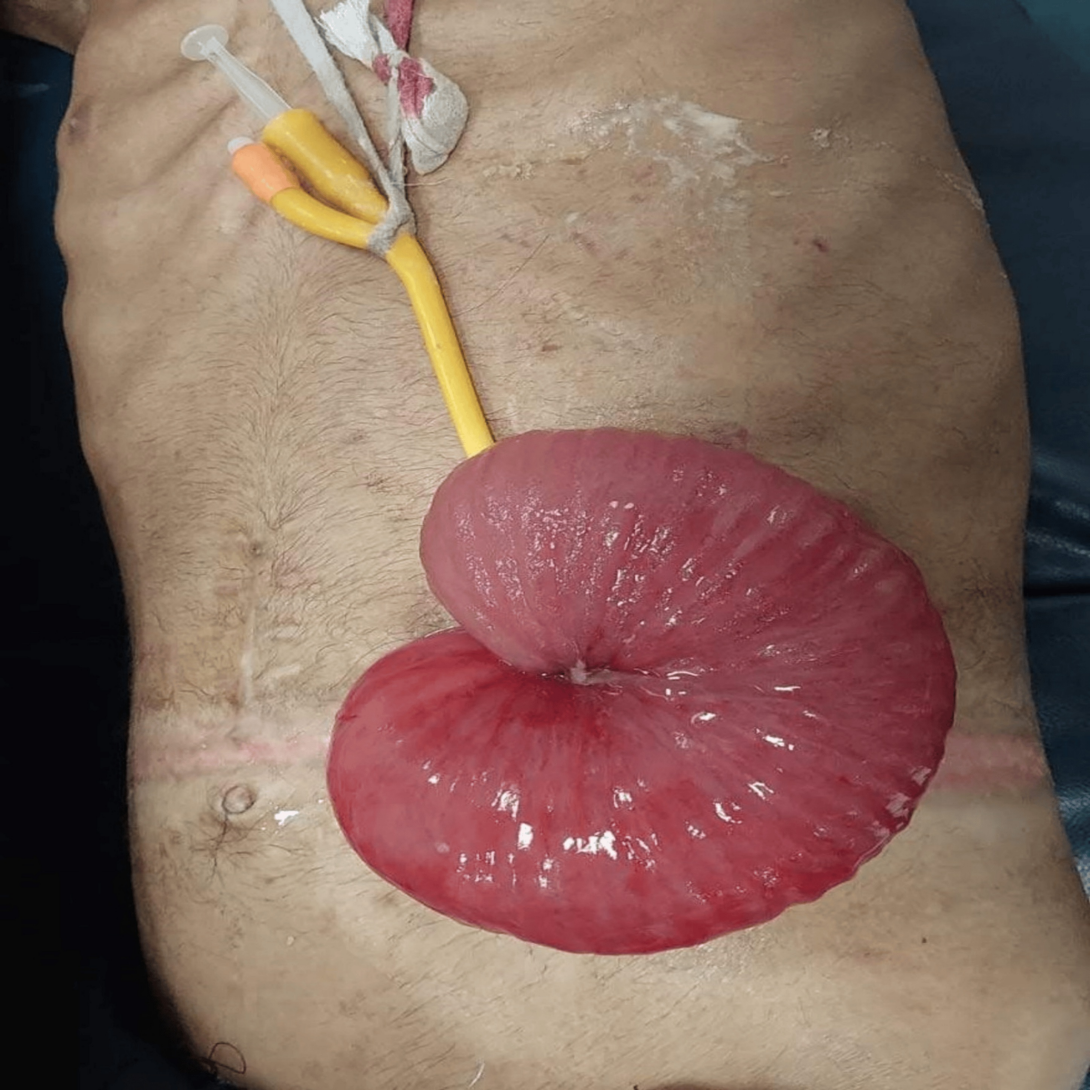
Clinical photo of the abdomen showing the initial clinical presentation. The dusky red, edematous, congested bowel rosette is clearly eviscerated through the previous FJ stoma, with the yellow catheter visible from the center. FJ: feeding jejunostomy

**Figure 2:**
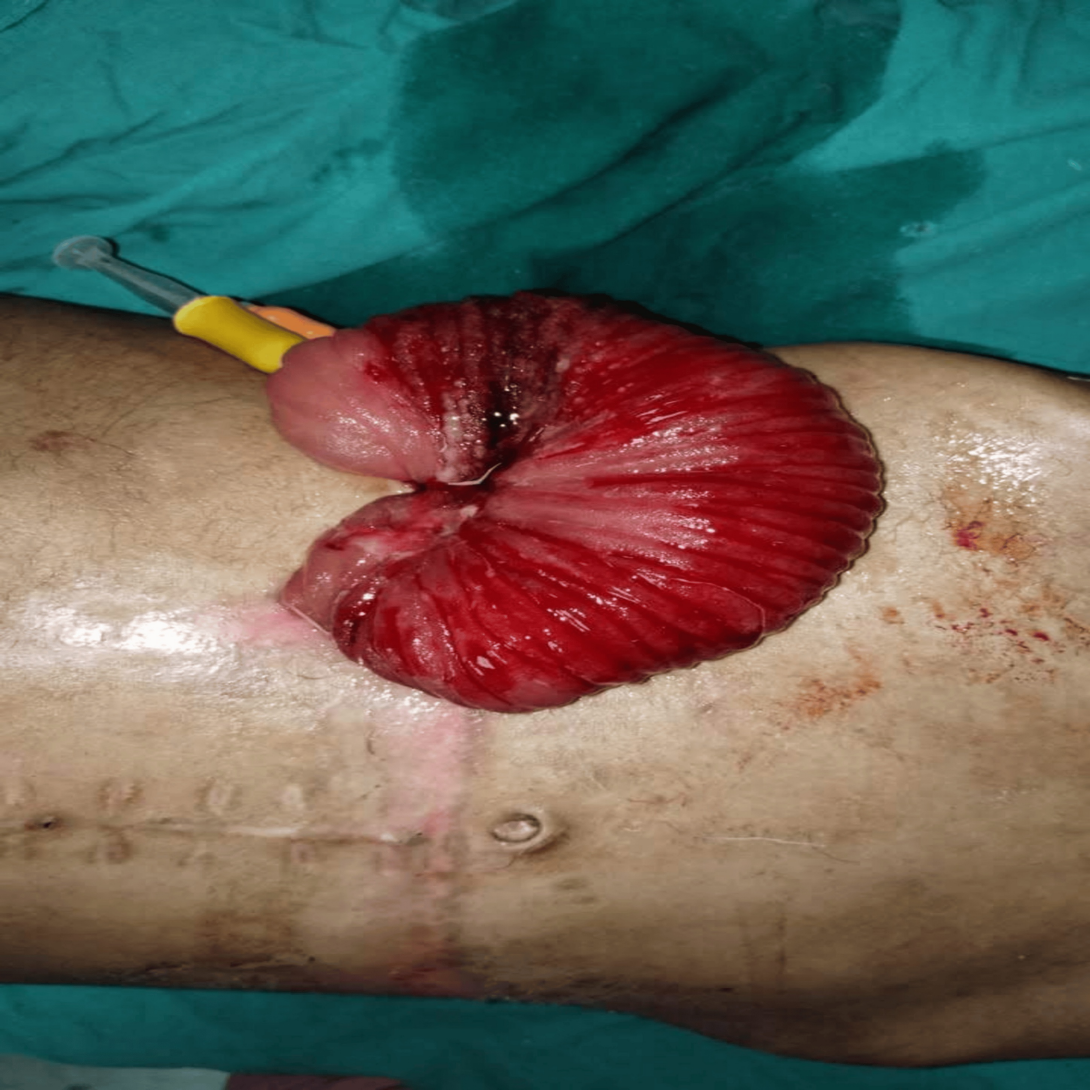
Close-up photo of the prolapsed jejunal bowel rosette on the anterior abdominal wall. It clearly shows the edematous mucosa and how the yellow catheter acted as the mechanical lead point.

On physical examination, the patient was found to have a large, edematous, and visibly congested rosette of eviscerated small bowel protruding through the anterior abdominal wall at the site of the established stoma. Strikingly, a yellow catheter, which had been utilized as the FJ tube, was seen exiting directly from the center of the prolapsed mucosal mass (Figures 1, 2). The surrounding anterior abdominal wall exhibited signs of local inflammation, and the patient had localized tenderness with guarding.

Given the acute evisceration and concern for bowel ischemia, the patient was resuscitated, routine blood work was sent, and the patient was immediately taken to the operating room for an emergency exploratory laparotomy.

**Figure 3:**
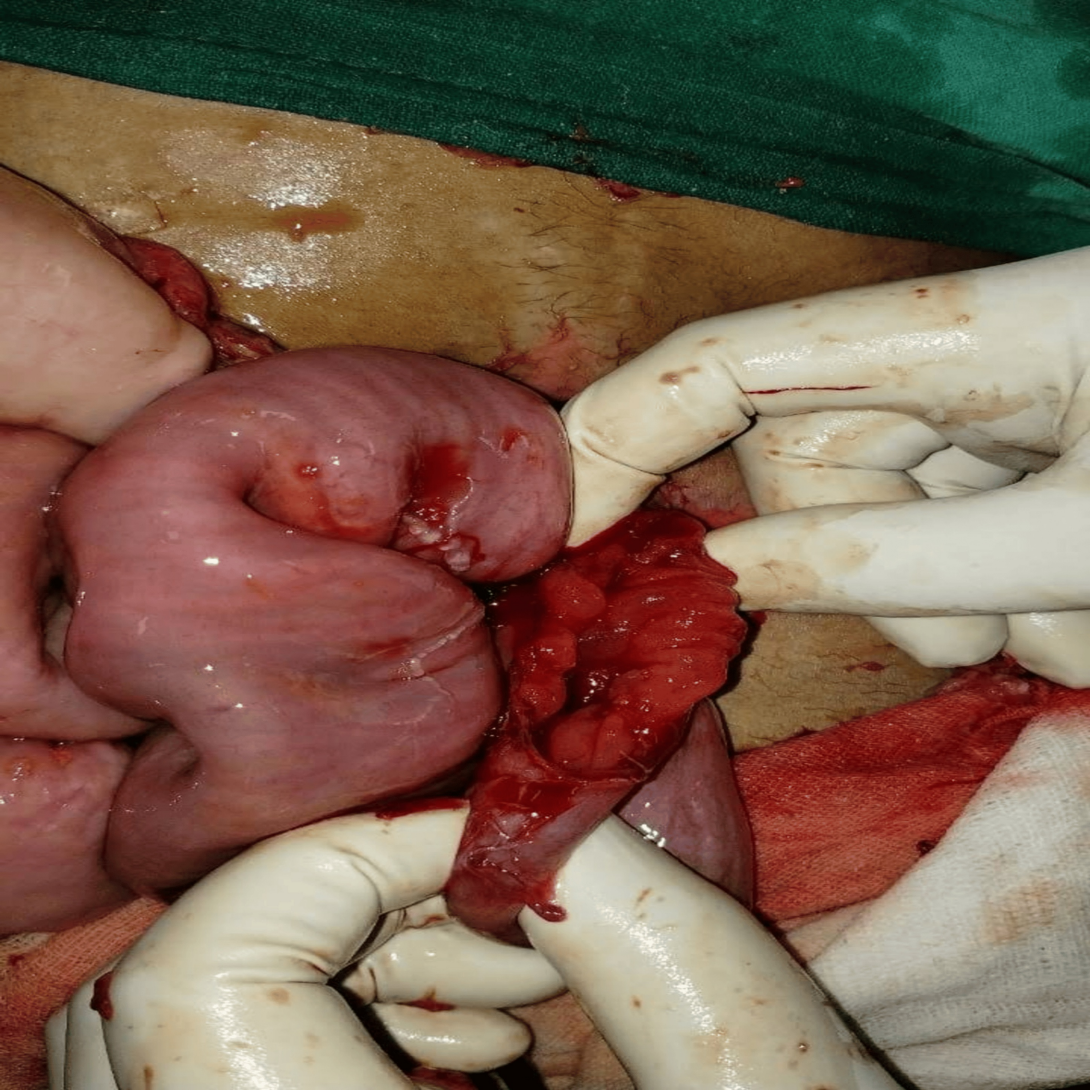
Intraoperative image of invaginating the intussuscepted segment, showing a perforation

Upon entering the abdomen via vertical midline laparotomy and meticulously mobilizing the involved segment, intraoperative findings confirmed a complex jejuno-jejunal intussusception acting as a lead point exactly at the site of the previous feeding jejunostomy. The involved bowel segment was significantly thickened, edematous, and displayed signs of vascular compromise. Upon complete reduction and inspection of the intussuscepted mass, a localized full-thickness perforation was identified on the jejunal wall adjacent to the tube insertion site (Figure 3).

**Figure 4:**
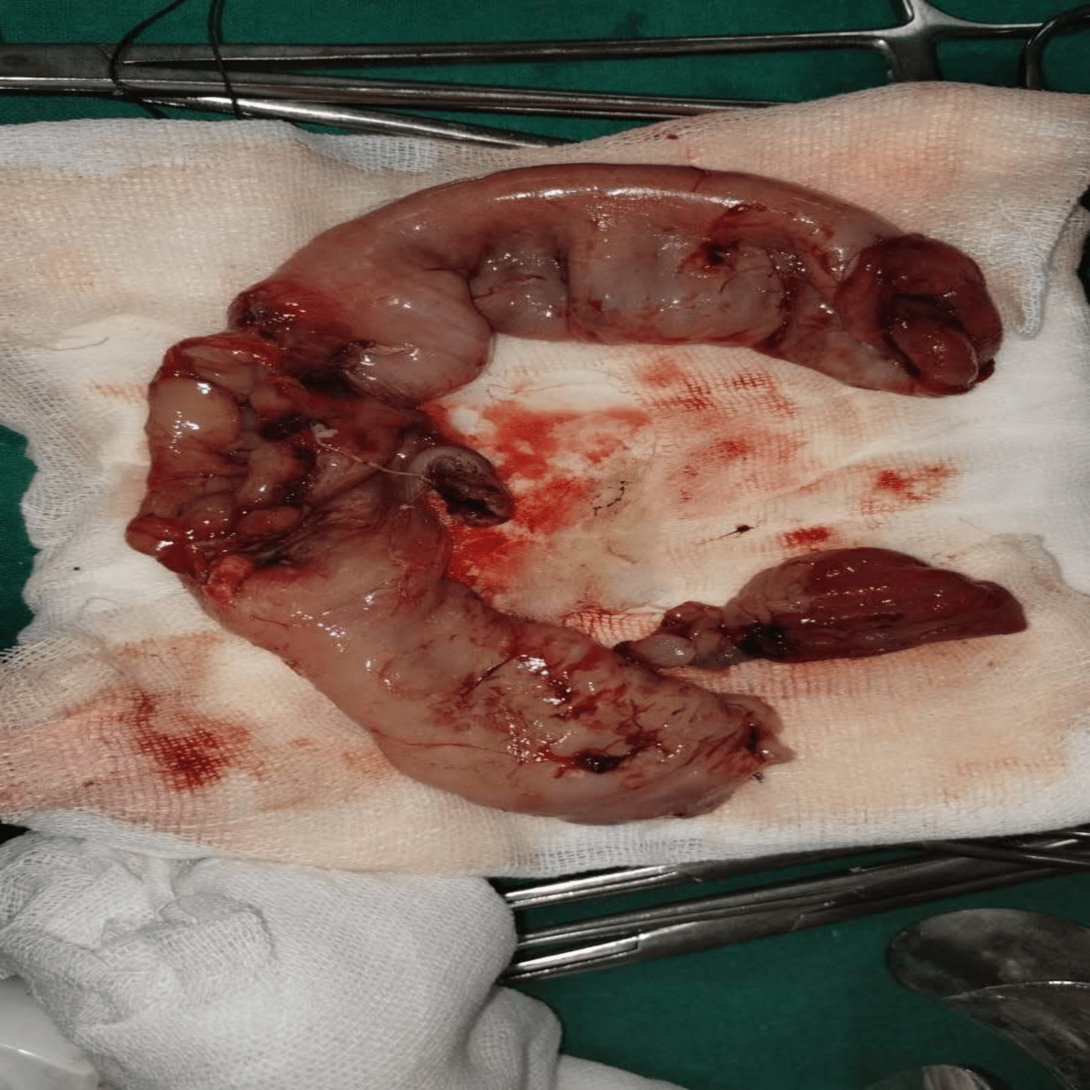
The gross resected segment of the compromised jejunum, including the area involved in intussusception and feeding jejunostomy site, laid out on the gauze.

Due to the presence of ischemia and gross perforation, bowel salvage of that specific segment was deemed unviable. A limited resection of the compromised jejunal segment containing the perforation and the intussusception complex was performed (Figure 4).

**Figure 5:**
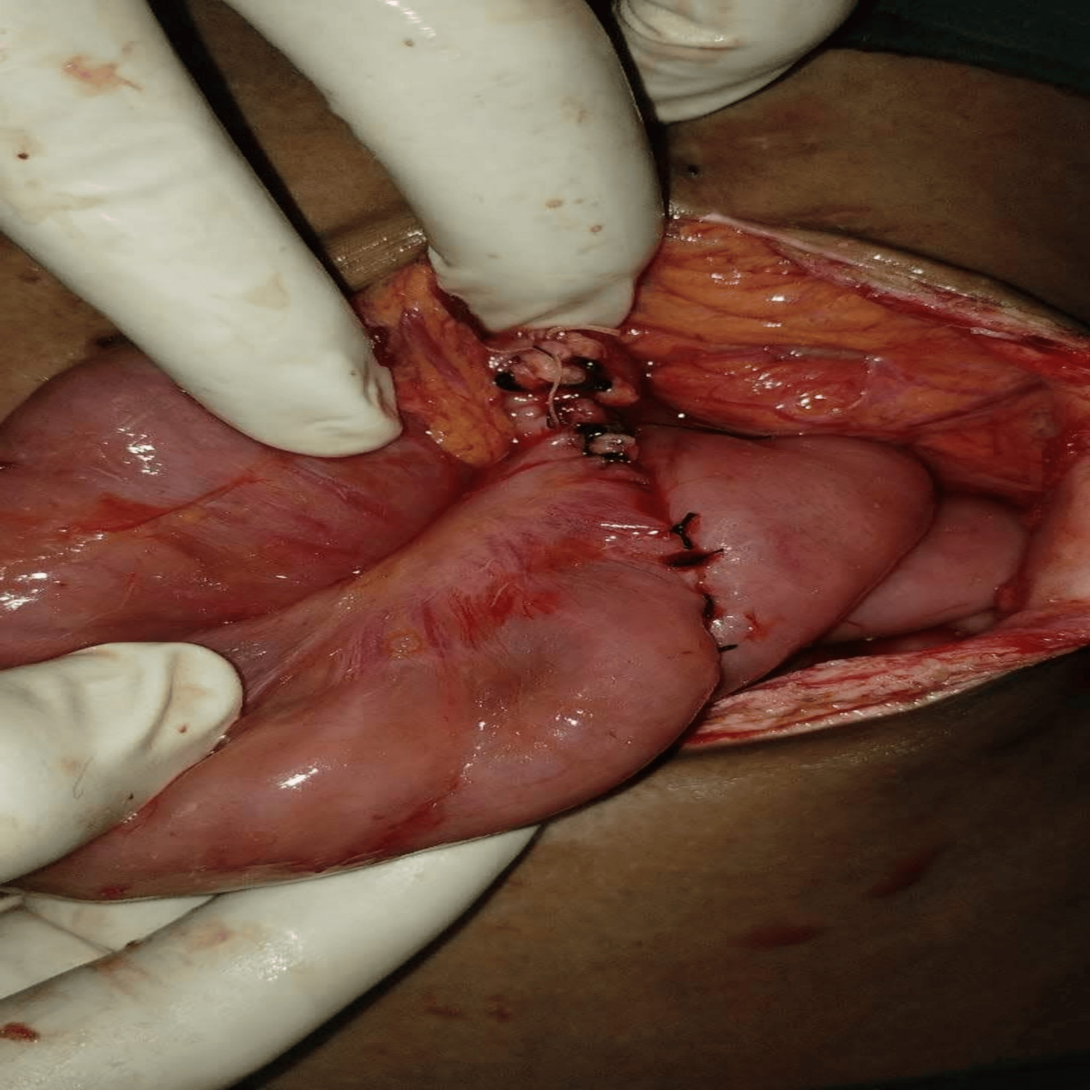
Newly created primary end-to-end jejuno-jejunal anastomosis after the resection.

Gastrointestinal continuity was successfully restored via a primary end-to-end jejuno-jejunal anastomosis (Figure 5). Recognizing the patient's ongoing need for enteral nutrition, a new FJ was carefully created in a healthy segment of the jejunum, safely distal to the newly fashioned anastomosis. Crucially, strict attention was paid to securing this new FJ segment to the anterior abdominal wall using an adequate seromuscular hitch stitch to prevent any future recurrence of prolapse or intussusception.

Following a thorough peritoneal lavage, the abdomen was closed. The patient's postoperative course was entirely uneventful. Enteral feeds through the newly established FJ were initiated gradually and were well tolerated. The patient remained hemodynamically stable, demonstrated normal bowel function, and was safely discharged on postoperative day 5.

## Discussion

Intussusception in adults is a rare clinical entity, accounting for a very small fraction of all mechanical bowel obstructions [3]. Because adult intussusception almost always has a definable structural lead point [4], an in situ FJ tube can easily become the culprit. This is especially true when a catheter, the "yellow tube" seen in our case, is used in resource-limited settings. The physical presence of the tube, the continuous infusion of feeds, or the inflation of the catheter balloon can disrupt normal peristalsis, triggering the proximal jejunal wall to telescope distally over the tube, with the inflated balloon often acting as the lead point, especially if the FJ segment is not fixed to the abdominal wall via hitch stitch. 

In our patient, the clinical picture was highly atypical and dramatic. The intussusception was accompanied by an external prolapse of the congested bowel directly through the stoma site, complicated further by a localized full-thickness perforation. This perforation likely resulted from progressive ischemia and pressure necrosis caused by the intussuscepted tissue complex compressing against the fixed anterior abdominal wall, or from direct mechanical trauma exerted by the tube itself.

Crucially, surgical technique plays a vital role in preventing such major mechanical complications. Proper fixation of the jejunal loop to the anterior abdominal wall using adequate seromuscular anchoring sutures, often referred to as a "hitch stitch", is a fundamental step to prevent abnormal bowel mobility [1]. Failure to properly anchor the bowel has been explicitly documented as a primary catalyst for severe mechanical complications, including closed-loop obstruction, volvulus, and intussusception [5,6]. This anchoring effectively holds the jejunum in place, preventing it from telescoping around the tube and significantly reducing the risk of external prolapse.

When these preventative measures fail or are inadequately performed, management of an eviscerated and intussuscepted bowel mandates emergent surgical intervention. In instances where signs of gross ischemia or perforation are present, as in this case, a limited bowel resection is required rather than a simple reduction. A primary anastomosis is generally safe provided the remaining bowel ends are healthy and well-perfused. Relocating a new FJ distal to the anastomosis, while ensuring proper abdominal wall fixation, allows for the early resumption of enteral feeding without placing tension on the new suture line.

## Conclusions

FJ is a crucial standard procedure for establishing long-term enteral nutrition, but clinicians must maintain a high index of suspicion for rare, life-threatening mechanical complications. We reported an exceptional case of FJ-induced jejuno-jejunal intussusception complicated by localized perforation and dramatic external evisceration, in which the "yellow tube" (catheter) acted as the mechanical lead point.

This case illustrates that an acute presentation of bowel evisceration around an existing stoma mandates immediate surgical exploration. Resection of the compromised bowel segment, primary anastomosis, and re-establishment of distal enteral access is a safe and effective management strategy. Crucially, strict adherence to meticulous surgical technique during the initial insertion, specifically securing the jejunal loop to the anterior abdominal wall using adequate anchoring sutures, is paramount to prevent abnormal bowel mobility and avert catastrophic complications like prolapse, volvulus, and intussusception.
